# Spatial and seasonal distribution of *Bulinus globosus* and *Biomphalaria pfeifferi* in Ingwavuma, uMkhanyakude district, KwaZulu-Natal, South Africa: Implications for schistosomiasis transmission at micro-geographical scale

**DOI:** 10.1186/s13071-021-04720-7

**Published:** 2021-04-23

**Authors:** Tawanda Manyangadze, Moses John Chimbari, Owen Rubaba, White Soko, Samson Mukaratirwa

**Affiliations:** 1 School of Nursing and Public Health, Department of Public Health Medicine, University of KwaZulu-Natal, Durban, South Africa; 2 Geography Department, Faculty of Science and Engineering, Bindura University of Science Education, Bag 1020, Bindura, Zimbabwe; 3 Ministry of Health and Child Care, De Beers Research Laboratory, P. O. Box 197, Chiredzi, Zimbabwe; 4 School of Life Sciences, University of KwaZulu-Natal, Durban, South Africa; 5 Center for Zoonoses and Tropical Veterinary Medicine, Ross University School of Veterinary Medicine, Basseterre, St Kitts and Nevis

**Keywords:** *Bulinus globosus*, *Biomphalaria pfeifferi*, *Schistosoma haematobium*, *Schistosoma mansoni*, Malacology, Ecology

## Abstract

**Background:**

Schsistosomiasis is endemic in sub-Saharan Africa. It is transmitted by intermediate host snails such as *Bulinus* and *Biomphalaria*. An understanding of the abundance and distribution of snail vectors is important in designing control strategies. This study describes the spatial and seasonal variation of *B. globosus* and *Bio. pfeifferi* and their schistosome infection rates between May 2014 and May 2015 in Ingwavuma, uMkhanyakude district, KwaZulu-Natal province, South Africa.

**Methods:**

Snail sampling was done on 16 sites once every month by two people for 30 min at each site using the scooping and handpicking methods. Snails collected from each site were screened for schistosome mammalian cercariae by the shedding method. The negative binomial generalised linear mixed model (glmm) was used to determine the relationship between abundances of the intermediate host snails and climatic factors [rainfall, land surface temperatures (LST), seasons, habitats, sampling sites and water physico-chemical parameters including pH and dissolved oxygen (DO)].

**Results:**

In total, 1846 schistosomiasis intermediate host snails were collected during the study period. *Biompharia pfeifferi* was more abundant (53.36%, *n* = 985) compared to *B. globosus* (46.64%, *n* = 861). *Bulinus globosus* was recorded at 12 sites (75%) and *Bio. pfeifferi* was present at 7 sites (43.8%). *Biompharia pfeifferi* cohabited with *B. globosus* at all the sites it was present. High numbers of *Bio. pfeifferi* (*n* = 872, 88.5%) and *B. globosus* (*n* = 705, 81.9%) were found between winter and mid-spring. Monthly rainfall showed a statistically significant negative relationship with the abundance of *B. globosus* (*p* < 0.05). Dissolved oxygen (DO) had a statistically significant positive relationship with the abundance of *Bio. pfeifferi* (*p* < 0.05) while (LST) had a statistically significant negative relationship (*p* < 0.05). More *B. globosus* (8.9%, *n* = 861) were shedding schistosome mammalian cercariae compared to *Bio. pfeifferi* (0.1%, *n* = 985) confirming the already documented high prevalence of *S. haematobium* in Ingwavuma compared to *S. mansoni*.

**Conclusion:**

Results of this study provide updated information on the distribution of schistosomiasis intermediate host snails in the study area and contributes towards the understanding of the transmission dynamics of schistosomiasis at the micro-geographical scale in this area.

**Graphic Abstract:**

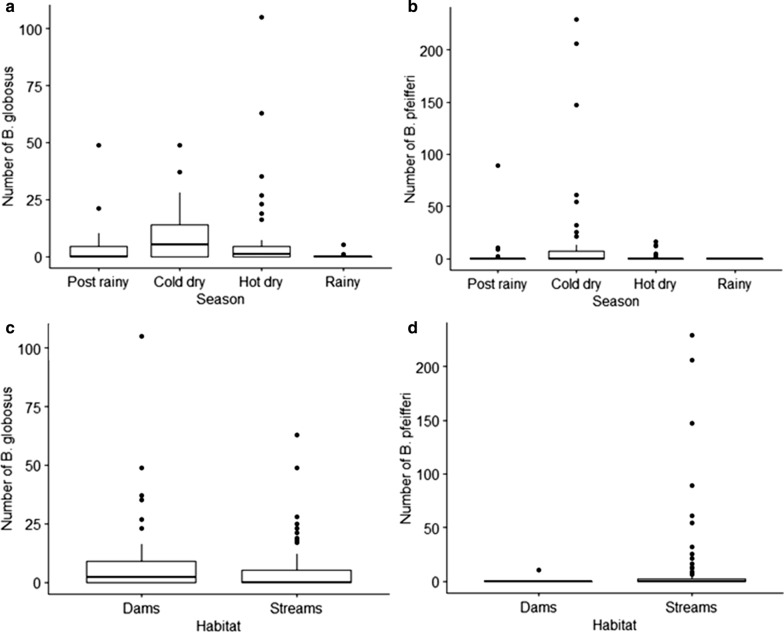

## Background

*Schistosoma haematobium* and *Schistosoma mansoni* are endemic in sub-Saharan Africa [1, 2] where they are mainly transmitted by *Bulinus globosus* and *Biomphalaria pfeifferi,* respectively [3, 4]. The transmission and focal distribution of schistosomiasis are spatially and temporally restricted to water bodies inhabited by its obligate intermediate host snails [1] and human water contact [5]. Schistosomiasis is a serious public health problem in South Africa. An estimated 2–3 million children are infected, and 20 million (nearly 40% of the population) are at risk [6]. Previous studies conducted in Ingwavuma of uMkhanyakude district of South Africa showed that the prevalence of *S. haematobium* was around 37.5% [7].

The density and abundance of the intermediate host snails for schistosomiasis are influenced by environmental and climatic factors. Environmental factors include physical and chemical water properties such as temperature, turbidity, salinity, conductivity, pH and velocity and biological factors such as the availability of food, competition, predator-prey interactions, presence and density of aquatic plants [1, 5, 8–10]. Rainfall and temperature have been reported as the main climatic factors that determine the distribution of intermediate host snails [4, 11, 12].

Although the afore-mentioned factors have been well studied, their importance varies from one ecological zone to another and even from one water body to another in the same ecological zone. The ecological factors influence the presence and abundance of intermediate host snails [13]. Thus, it is important to conduct studies at a local scale to identify factors that are significant in particular habitats to understand the schistosomiasis transmission dynamics [14]. Information on the ecology, temporal distribution and population dynamics of *B. globosus* and *Bio. pfeifferi* at a local scale is needed to enhance the knowledge of the spatial and seasonal distribution and factors influencing these patterns [11]. This provides critical data for the development and optimization of control and management of the disease [15]. Although much work has been done to unravel the limiting factors for snail habitat preferences [11, 12, 16], it is important to gather data at a micro-geographical scale to understand the local transmission dynamics [11, 16, 17].

The aim of this study was to describe and analyse the spatial and seasonal variation of *B. globosus* and *Bio. pfeifferi* in relation to a set of ecological factors at a micro-geographical scale in Ingwavuma of uMkhanyakude district, KwaZulu-Natal province, South Africa. Sound knowledge of the abundance and distribution of intermediate host snails is key to understanding schistosomiasis transmission in order to design effective intervention strategies in endemic areas [9].

## Methods

### Study area

**Fig. 1:**
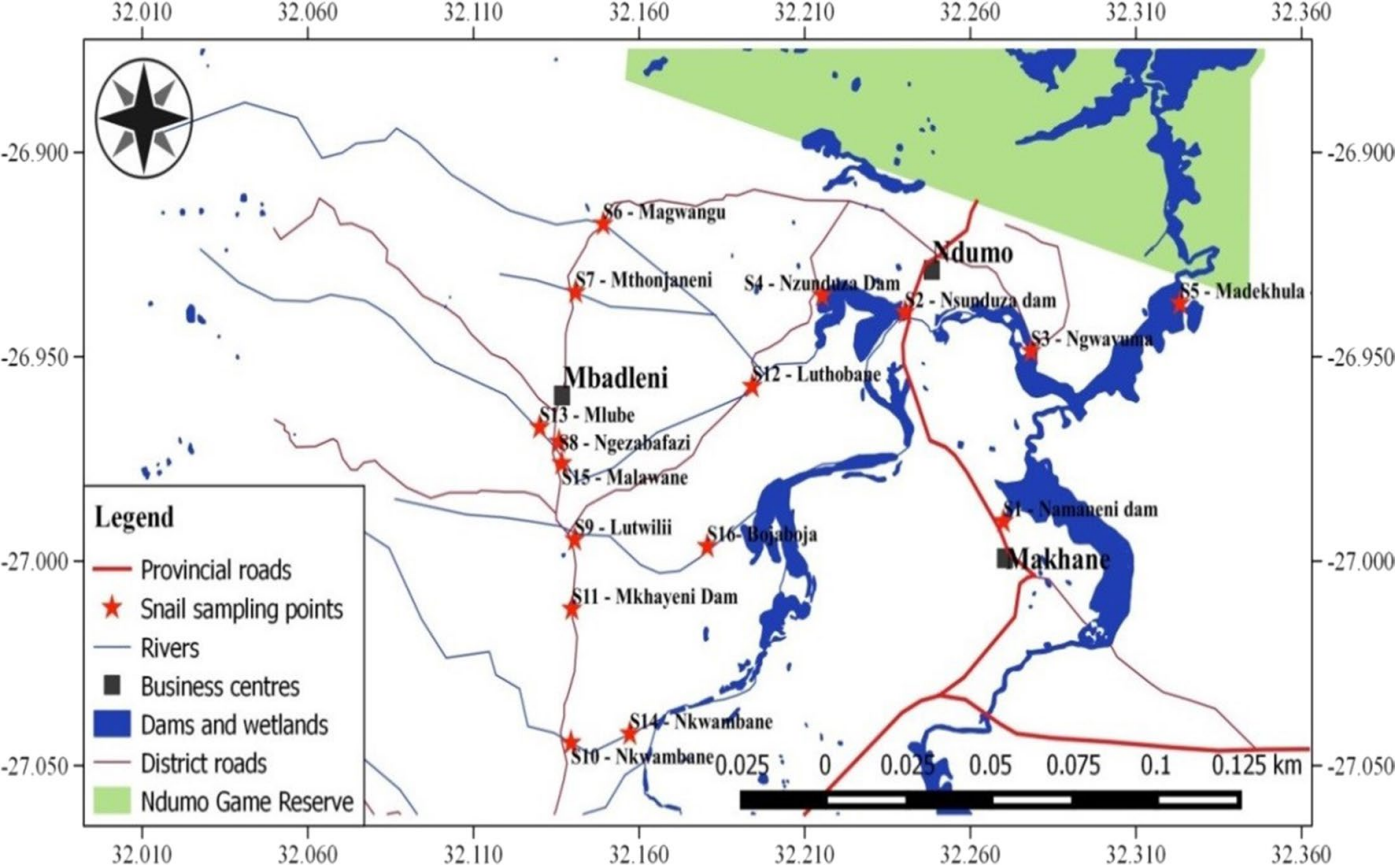
Study area and snail sampling sites (indicated by red star) in Ingwavuma (Ndumo area), uMkhanyakude District, KwaZulu-Natal, South Africa (May 2014 to May 2015) (Adapted from Manyangadze et al., [11])

The study was conducted in Ingwavuma (Fig. 1) of uMkhanyakude Health District in KwaZulu-Natal (KZN) province, South Africa, between May 2014 and May 2015. uMkhanyakude district is located in the northernmost eastern part of KwaZulu-Natal province of South Africa bordering Mozambique and Swaziland to the north and northwest respectively. The climate ranges from tropical to subtropical [18] with a hot wet summer and a cold dry winter. uMkhanyakude experiences low annual rainfall averaging 690 mm per year. More than half of the households in this area do not have access to piped clean water and have poor sanitary facilities [19, 20] making them vulnerable to water-borne diseases (WBDs) including schistosomiasis. The study area is approximately 40 × 30 km and mostly characterised by seasonal streams flowing towards the Pongola flood plain and two main rivers (Pongolo and Ngwavuma) and there are two major dams—Nsunduza and Namaneni (Fig. 1).

### Surveys for schistosomiasis vector snails

*Bulinus globosus* and *Bio. pfeifferi* snails were sampled from 16 sites distributed across the whole study area (Fig. 1) every month from May 2014 to May 2015. Sampling sites (including streams and dams) were identified based on observations on human water contact [21] and following Appleton and Miranda’s [6] advice that snails need to be collected from suspected transmission sites and screened for infection for the purposes of local schistosomiasis surveillance. Twelve of the sites were located at pools along the seasonal streams and 4 sites were located at dams to capture the spatial variation in snail population dynamics at human water contact patterns and different habitats. The snail sampling was semi-quantitative and was conducted by the same two technicians using scoops made from a kitchen sieve mounted on a broom stick [22, 23] and handpicking live visible snails for 30 min on each site [6]. Snails collected were morphologically identified [6] and expressed as number of snails.

### Cercarial shedding from host snails

Snails collected from the study sites were identified as described by Brown [4] and screened for schistosome infection by the shedding method [24]. Briefly, snails were placed in small glass tubes containing 10 ml of filtered pond water as described by Chimbari et al. [25] and exposed to strong artificial light to induce cercarial shedding [24]. Emerging cercariae were inspected under a dissecting microscope at 300× magnification [6, 21] and identified as described by Frandsen and Christensen [26]. *Bulinus globosus* and *Bio. pfeifferi* were designated as infected with mammalian schistosome cercarie if they shed bifurcated cercariae. The number of infected snails for each species at each site per month was recorded and expressed as a percentage [27].

### Environmental and climatic factors

The environmental and climatic data were obtained through field measurement and remote sensing. Water temperature, pH, salinity and conductivity were measured using a portable water meter (Hanna Instruments, Møllevænget, Sweden). Remote sensing data including the climatic data including rainfall from Climate Hazards InfraRed Precipitation (CHIRPS), minimum and maximum land surface temperature (LST)—Moderate Resolution Imaging Spectroradiometer (MODIS) were downloaded through the International Research Institute for Climate and Society (IRI) data library (http://iridl.ldeo.columbia.edu/SOURCES/). The data on water physico-chemical parameters were gathered simultaneously with snail surveys. Climatic data were aggregated to monthly totals (rainfall) and monthly averages (temperature). These data were used to determine the relationship between the climatic factors and snail distribution by month, site, season and habitat. The year was divided into four seasons according to temperature and rainfall: rainy (December to February), post-rainy (March to May), cold-dry (June to August) and hot-dry (September to November) based on previous studies in the same area [11].

### Statistical analysis

Negative binomial generalized linear mixed models (GLMMs) were used to model the abundance of *B. globosus*, *Bio.pfeiferi* and shedding *B. globosus* in relation to climatic factors, water physico-chemical properties, habitat types (i.e. dams and streams) and seasons with sites as random effects to ensure that intra-site correlation is accounted for in the parameter estimates. The models were fitted using the ‘glmmTMB’ [28] package in R Version 3.6.1. The collinearity and relationship between variables were checked using the variance inflation factor (VIF). Variables with VIF > 5 indicate multicollinearity [29] and hence were not considered in the current analysis.

## Results

### Spatial and seasonal variation of *Bulinus globosus* and *Biomphalaria pfeifferi* abundance

**Fig. 2:**
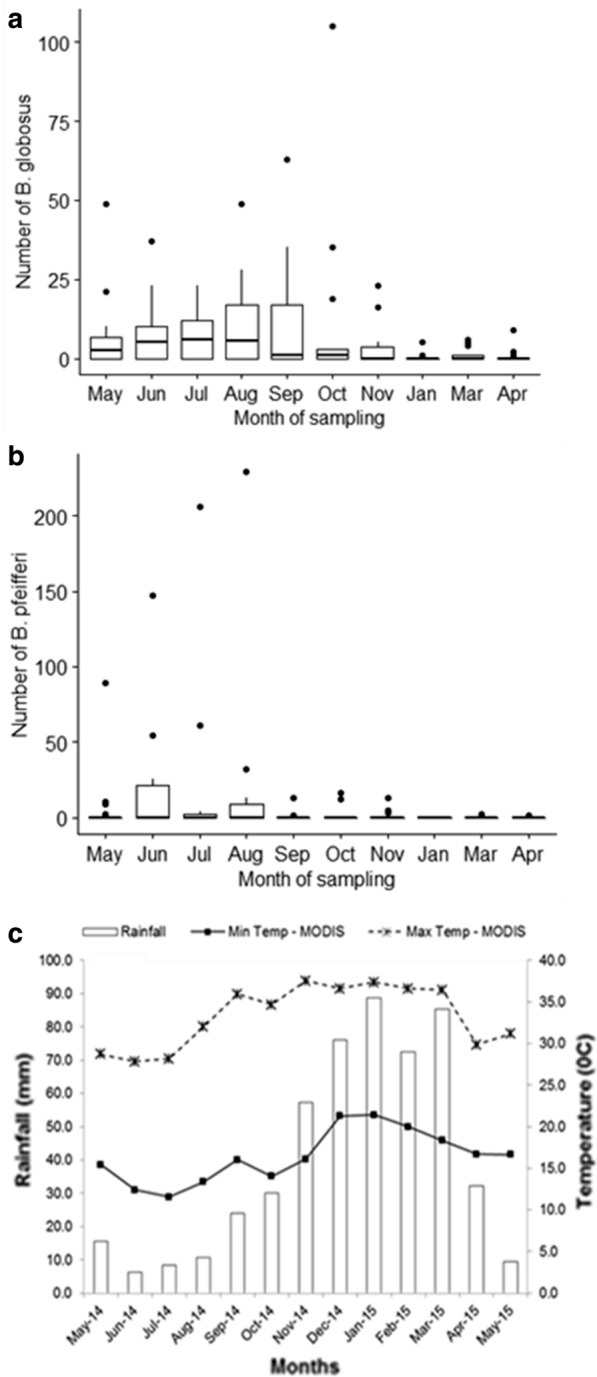
Number of *Bulinus globosus* collected by month (**a**). Number of *Biomphalaria pfeifferi* collected by month (**b**) and monthly average rainfall and average minimum and maximum temperature in in Ingwavuma (Ndumo area) from May 2014 to May 2015 (**c**). Snails were collected by two men for 30 min once every month. For box and whisker plots in **a** and **b** the box represents the lower quartile (0.25), the median (0.5) and the upper quartile (0.75); the whiskers show the variability outside the box, i.e. the minimum excluding outliers and maximum also excluding outliers and outliers are shown as individual points

**Fig. 3:**
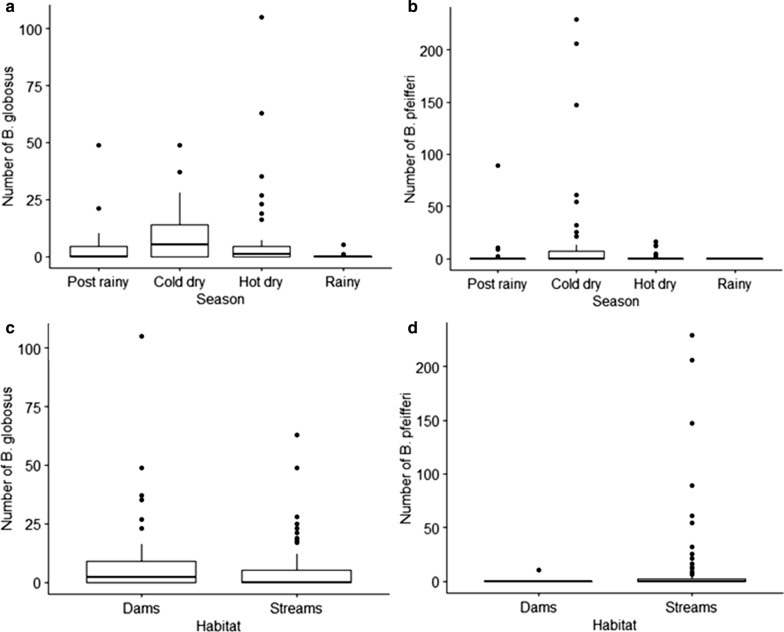
Box and whisker plots showing **a** the number of *B. globosus* collected by season, **b** number of *Bio. pfeifferi* collected by season, **c** number of *B. globosus* collected by habitat type and **d** number of *Bio. pfeifferi* collected by habitat type. Snails were collected by two men for 30 min once every month. In all figures, the box represents the lower quartile (0.25), the median (0.5) and the upper quartile (0.75); the whiskers show the variability outside the box, i.e. the minimum excluding outliers and maximum excluding outliers and outliers are shown as individual points

In total, 1846 schistosomiasis intermediate host snails were collected during the study period (May 2014 to May 2015). *Biompharia pfeifferi* was more abundant (53.36%, *n* = 985) compared to *B. globosus* (46.64%, *n* = 861). However, *B. globosus* was found at more sites (12, *n* = 16) compared to *Bio. pfeifferi* (7, *n* = 16). *Bio. pfeifferi* cohabited with *B. globosus* at all sites it was present. Most of the *Bio. pfeifferi* snails (99%) were recovered from stream habitats while about 52.4% of *B. globosus* were recovered from stream habitats (Fig. 3c, d). High *B. globosus* counts were recorded from the end of the cold-dry season (August) to the mid hot-dry season (October 2014) and the counts decreased from November 2014 to May 2015 (Fig. 2a).

The climatic factors (rainfall, minimum temperature and maximum temperature) for Ingwavuma are shown in Fig. 2c. The variation in snail abundance by seasons is shown in Fig. 3a, b. High counts of *Bio. pfeifferi* were found during the cold-dry season (June to August 2014) with a sharp decrease from the beginning of the hot-dry season—September 2014 to May 2015 (Fig. 3a, b).

The summary of properties of the climatic and environmental exploratory variables for *B. globosus* from negative binomial regression in “*glmmTMB*” is shown in Table 1. Monthly rainfall showed a statistically significant negative relationship with the abundance of *B. globosus* (*p*-value < 0.05) while other variables did not show any statistically significant relationship (Table 1).

**Table 1:** Summary of properties of climatic and environmental exploratory variables for *Bulinus globosus, Biomphalaria pfeifferi* and shedding *B. globosus* from negative binomial regression GLMM in “glmmTMB package in R3.6.1”

Species	Fixed variables	Estimate	Confidence interval (CI)	*p*-value	Random effects^a^	
					Variance	Standard deviation
*Bulinus* *globosus*	Intercept	2.727	− 1.976 to 7.430	0.256	2.069	1.438
	Rainfall	− 0.023	− 0.043 to − 0.003	0.022*		
	Minimum LST	0.206	− 0.470 to 0.058	0.126		
	Maximum LST	0.071	− 0.086 to 0.228	0.374		
	Water pH	− 0.1235	− 0.471 to 0.201	0.452		
	Dissolved oxygen	0.007	− 0.001 to 0.016	0.076		
	Habitat—streams	− 0.210	− 1.979 to 1.559	0.816		
	Season—hot dry	0.475	− 0.490 to 1.441	0.335		
	Season—post rainy	0.155	− 1.077 to 1.387	0.806		
	Season—rainy	0.871	− 1.800 to 3.541	0.523		
*Biomphalaria* *pfeifferi*	Intercept	6.126	− 2.980 to 15.233	0.187	10.22	3.197
	Rainfall	0.012	− 0.016 to 0.039	0.410		
	Minimum LST	− 0.535	− 0.933 to − 0.138	0.008*		
	Maximum LST	− 0.023	− 0.202 to 0.157	0.804		
	Water pH	− 0.506	− 1.097 to 0.087	0.094		
	Dissolved oxygen	0.015	0.003 to 0.026	0.012*		
	Habitat—streams	4.214	− 0.323 to 8.751	0.069		
	Season—hot dry	− 0.923	− 2.581 to 0.736	0.275		
	Season—post rainy	− 0.205	− 1.726 to 1.317	0.792		
	Season—rainy	− 16.72	− 150.11 to 149.36	0.998		
Shedding *Bulinus* *globosus*	Intercept	− 5.361	− 13.219 to 2.498	0.181	3.229	1.797
	Rainfall	− 0.022	− 0.066 to 0.022	0.321		
	Minimum LST	− 0.298	− 0.856 to 0.259	0.295		
	Maximum LST	0.134	− 0.167 to 0.436	0.384		
	Water pH	0.412	− 0.084 to 0.907	0.103		
	Dissolved oxygen	0.011	− 0.003 to 0.025	0.109		
	Habitat—streams	− 0.083	− 2.608 to 2.441	0.948		
	Season—hot dry	− 0.645	− 2.256 to 0.965	0.432		
	Season—post rainy	− 0.086	− 2.746 to 2.574	0.950		
	Season—rainy	1.624	− 3.413 to 6.661	0.527		

*LST* Land surface temperature

*Significant at *p* < 0.05

^a^Sampling sites was the random effect. Reference category for seasons was cold dry season and for the habitats it was dams

Only DO showed a statistically significant positive association with the abundance of *Bio. pfeifferi* (*p* < 0.05) (Table 1). Minimum LST had a statistically significant negative association with the abundance of *Bio. pfeifferi* (*p* < 0.01). Other variables did not show a statistically significant relationship with the abundance of *Bio. pfeifferi* (Table 1).

### Spatial and seasonal variation of shedding *Bulinus globosus* and *Biomphalaria pfeifferi* abundance

**Fig. 4:**
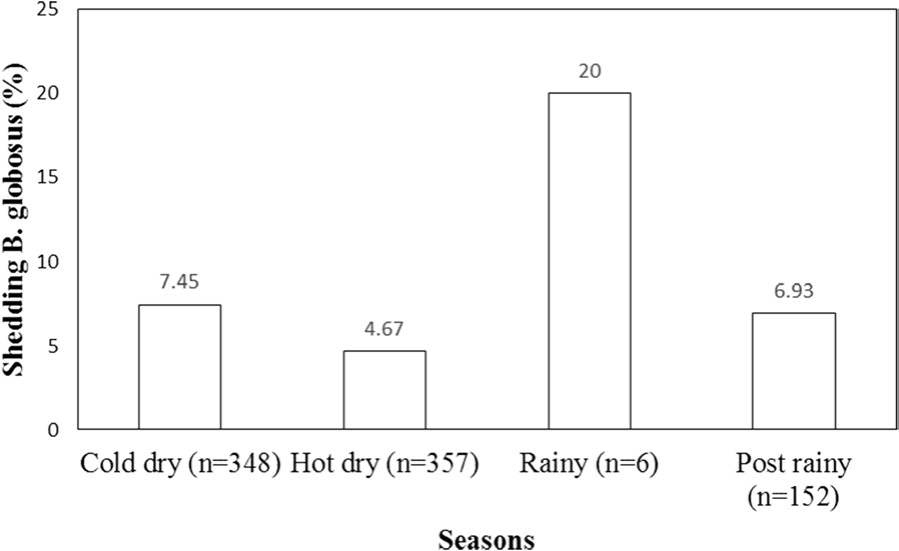
Seasonal proportion (%) of shedding *Bulinus globosus* in Ingwavuma (Ndumo area) area, uMkhanyakude, South Africa, from May 2014 to May 2015

*Bulinus globosus* showed a continuous pattern of shedding mammalian schistosomes by seasons (Fig. 4) and *Bio. pfeifferi* only shed in August 2014 (0.22%). Therefore, more *B. globosus* (8.9%, *n* = 861) were shedding mammalian cercaria compared to *Bio. pfeifferi* (0.1%, *n* = 985). More *B. globosus* snails were infected in the rainy season (20%) as shown in Fig. 4. None of the variables considered in this study showed a statistically significant relationship with abundance of shedding *B. globosus* based on the negative binomial GLMM (Table 1). Although more shedding *B. globosus* were collected during the rainy season than in other seasons (Fig. 4), the difference was not statistically significant (negative binomial GLMM; Table 1).

## Discussion

Our findings on the presence of *B. globosus* and *Bio. pfeifferi* corroborate with those of N’Guessan et al. [30] and Mohammed [31]. We observed that snail abundance varied by sites and habitats indicating the focal nature of schistosomiasis. Monthly and seasonal variation was also observed. Snails tend to breed intensively and establish large populations following the onset of rains [32]. Populations of *B. globosus* in inland sites are rather erratic, mainly found during and a few months after the rainy season [33].

Rainfall was negatively associated with both *B. globosus* and *Bio. pfeifferi*. However, that relationship was only stronger at a significant level for *B. globosus*. An association among snail distribution, abundance and rainfall has also been demonstrated in several studies. The condition of snail habitat is affected by rainfall in diverse ways. Snails cannot survive without water but too much water also reduces snail populations [34]. Rainfall contributes to the creation of temporary snail habitats and also supports creation of new habitats as there is transportation of snails by heavy rainfall. However, rainfall may also sharply reduce population densities [1] as the speed of the flowing water > 0.3 m/s might be a limiting factor for snail abundance [35]. We did not measure the water velocity, an important parameter in elucidating the complex relationship between water velocity and vector snail density. During the post-rainy season and cold and dry seasons, the pools (along the streams) and dams provided suitable habitats for both *B. globosus* and *Bio. pfeifferi* as observed by Utzinger and Tanner [36] and [5]. However, in the hot dry season most of the rivers were dry; hence, few snails were found as they might have been aestivating as suggested by Betterton [37] and Rubaba et al. [38]. Thus, the shortage of surface water might be the main limiting factor for the abundance and density of snails in this particular area during the study period. Therefore, long dry periods that may be induced by climate change may result in fewer snails but there are also chances for the surviving snails to repopulate the habitats [38]. Further investigations during periods of normal rain might elucidate the relationship among rainfall, surface water levels and snail population densities and abundance. We have previously demonstrated that the availability of surface water (as represented by Normalised Difference Water Index) has a higher contribution in determining the spatial distribution of snail habitat in the same area based on the Maxent model [11]. However, according to Brooker [39], the spatial relationship between rainfall and snail population dynamics and infection transmission is difficult to measure since the effect of rainfall varies depending on the species of snail and the geographical location. Where snails occur in streams, as in the present study area, rainfall plays an important role in reducing snail populations and the population “starts afresh” after each rainy season, but the effect is only temporary as repopulation occurs rapidly [21].

The monthly *Bio. pfeifferi* abundance had a significant negative association with minimum land surface temperature. Temperature has also been identified as one of the key factors that influence the distribution of aquatic organisms, as it can be limiting for *Bio. pfeifferi*, when both high or low [40]. Snails have been observed to tolerate temperatures between 20 and 27 °C [41]. Temperatures lower than 20 °C tend to reduce breeding. *Biomphalaria pfeifferi* snails are less tolerant of higher temperatures and have not been observed where temperatures exceed 27 °C for more than 120 h per week but Bulinid snails seem to be better adapted to higher temperatures [14].

*Biomphalaria pfeifferi* showed high sensitivity to changes or differences in pH and DO compared to *B. globosus*. Kazibwe et al. [42] and Chimbari et al. [10] also noted the role played by these factors in the distribution of these snail species. Previous models have demonstrated that pH values and other physicochemical properties affect spatial distribution and snail densities [5, 43].

The percentage of infection in intermediate host snails could act as a composite index of both miracidial and cercarial densities [44] indicating the transmission of schistosomiasis in that particular area and time. *Bulinus globosus* snails had higher infection rates compared to *Bio. pfeifferi,* suggesting that *S. haematobium* is more prevalent than *S. mansoni* in Ingwavuma. Although we did not identify cercariae to the genus level, it has been observed that the species of snail from which schistosome cercariae are shed give a clue to their identity. *Schistosoma haematobium* cercariae are shed by *B. globosus* (and also *B. africanus*) and *S. mansoni* cercarie are shed by *Bio. pfeifferi* [6]*.* It was also noted that all the sites in this study were accessible to people and cattle. Appleton and Miranda [6] noted that it is helpful to keep notes on the usage of water bodies by both people and domestic animals as *S. mattheei* cercariae can also develop in *B. globosus* (and also *B. africanus*). Therefore, it is possible that there is *S. mattheei* from *B. globosus.* However, our results show that *S. haematobium* is prevalent as reported in previous prevalence survey studies [7, 45]. This indicates that malacological surveys can complement the disease prevalence surveys in determining or planning of the control and management of schistosomiasis at micro-geographical scales. The results of this study contribute to knowledge on the dynamics of *S. haematobium* in Ingwavuma in support of Kabuyaya et al. [7], Manyangadze et al. [11], Saathoff et al. [45] and Manyangadze et al. [46]. Saathoff et al. [45] noted that in uMkhanyakude bilharzia is transmitted in summer to children engaged in recreational activities and our study has indicated that *B. globosus* snails were shedding cercarie in all seasons with the highest proportion in the rainy season (summer). However, we managed to collect samples in one month only in the rainy season (January 2015); hence, this may not be representative of the whole season. The presence of schistosome-infected *B*. *globosus* snails confirms that some of the sites considered in this study are *S. haematobium* transmission sites. The presence of uninfected *B. globosus* and *Bio. pfeifferi* snails at some sites indicates the sites’ potential for transmission of both *S. haematobium and S. mansoni*. The infection rates from our study confirm the observation by Appleton and Miranda [6] who noted that snail infection rates are commonly between 2 and 10%, but in some situations may be as high as 50%. The same authors emphasised that finding even one infected snail in a water body is evidence of schistosomiasis transmission.

During the period of this study, there was no snail control or surveillance programmes in Ingwavuma. Therefore, the results of this study provide useful information on snail distribution and abundance for managing schistosomiasis at the micro-scale. As also noted by Olkeba et al. [47], the information regarding the spatial and temporal distribution of schistosomiasis intermediate host snails can be used for development and implementation of effective snail control programmes to complement other schistosomiasis control initiatives such as mass drug administration.

## Conclusion

This study showed that *B. globosus* had a wider distribution compared to *Bio. pfeifferi*. *B. globosus* was found at more sites compared to *Bio. pfeifferi* but co-habitation of the two species was evident. *Bulinus globosus* numbers significantly inversely correlated with rainfall. Minimum land surface temperature was a significant limiting factor for *Bio. pfeifferi* abundance while DO had a significant positive correlation with *Bio. pfeifferi*. *Bulinus globosus* snails had higher infection rates compared to *Bio. pfeifferi,* indicating that *S. haematobium* is more prevalent than *S. mansoni* in Ingwavuma. This study provides updated information on distribution of schistosomiasis intermediate host snails in the study area and contributes towards the understanding of transmission dynamics of schistosomiasis at the micro-geographical scale in this area.

## Data Availability

The data supporting the conclusions of this article are included within the article.

## Abbreviations

CHIRPS: Climate Hazards Group InfraRed Precipitation with Station data
IRI: International Research Institute for Climate and Society
MODIS: Moderate resolution imaging spectroradiometer
LST: Land surface temperature
DO: Dissolved oxygen
CI: Confidence interval
GLMM: Generalized linear mixed models
AIC: Akaike’s information criterion
